# Pulmonary Congestion and Its Treatment.—I

**Published:** 1895-04-27

**Authors:** Benjamin Ward Richardson


### Pulmonary Congestion and Its Treatment.—I.

By Sir Benjamin Ward Richardson, M.D., F.R.S.

From the various observations made in the previous papers in this journal, all bearing upon the lesser circulation, I am naturally led to consider the treatment of pulmonary congestion. If nothing had been said bearing upon the physics of this question, sufficient has arisen in the last two months to attract practical attention. In the late epidemic we have been doing very little more than meet the symptoms of congestion of the lungs, congestion being almost synonymous with what is called influenza. I have not met with a single case of the disease that could be considered of moment, certainly that could be considered dangerous, without finding some evidence of pulmonary congestion. I have found signs of congestion in several instances where I did not expect it; the general symptoms not giving an indication of the mischief that was going on in the chest, so that after a little time whether the symptoms seemed grave or not, I made it a point to examine carefully the lungs of all the patients who came before me either in private or hospital practice. The result was, as I have stated, an invariable discovery of some form of derangement in the lesser circulation. There were days in last February in which I believe every patient under my care in hospital showed some indication of the disturbance popularly called influenza, and whenever they did so complain there was evidence of pulmonic derangement, so uniform seemed the influence at work in producing the abnormal conditions that promoted the disorder. It was remarkable how sometimes these conditions were extreme, and how quickly they passed away. I had a patient in whom the congestion appeared to extend over the whole pulmonic surface; the temperature, 104 deg., the pulse 100, and extremely tense; the action of the heart laboured; the mind obscured almost to coma. I had a strong inclination to take blood from this patient, but waited, with the determination that should pain occur or the coma become complete, I would certainly resort to venesection. To my surprise, in a few hours everything that might be considered dangerous subsided as if by a natural effort of recovery, and convalescence was established without any exercise of art except attendance to some rules which have to be described.

What then are the rules most proper for therapeutical purposes?

If it be clearly proved that the lungs are subject to congestion, whether there be, or be not, effusion, how ought we to treat?

I believe still that in the acute stage the old-fashioned remedy, extraction of venous blood, is sound, and the success which I remember to have seen in my early days following upon it in such cases, favours this idea. I well remember when we never hesitated to bleed in congestion of the lungs. The treatment was looked upon as being one of the canons of medicine, and a man would have been "ploughed" for ignorance on this point. Furthermore, I never saw any danger from the practice, either in young, middle-aged, or old. I can well recall bleeding children. I recollect a case of extreme congestion, with a pleuritic rub and acute pain, occurring in a young man, a tailor by trade, and a very emaciated-looking subject. He had so much pain in the chest, where the rub was most audible, that he found no rest, and was lying, when I arrived, across the board upon which he usually sat at work, face downward. He was pale from suffering. I placed him in a Windsor chair without the slightest hesitation as to what ought to be done, and notwithstanding his faintness drew blood. The blood at first was very dark, but as it continued to flow it grew of redder colour, just as Hippocrates observed it so to change in his day. When the venous blood had become almost arterial in character, and a pint had been drawn, the man seemed literally cured. The pain disappeared, the pleuritic rub was gone, the breathing was free, the skin moist, and the countenance of much better colour. But the patient was not allowed to resume work; he was recommended to take complete rest, and spend most of his time in bed for a couple of days. He had also an effervescing saline mixture, as it was called, and at night a calomel and rhubarb pill. Within a week he was at his work again, and as well as ever. Here was an instance in which extreme congestion of lung was immediately relieved, together with the right side of the heart, which had begun to fail from the burden thrown upon it, by removing blood. Had it not been relieved, stasis would probably have commenced on the right side of the heart, and in the lungs; then the struggling right heart, failing to send over its proper complement of blood to the left side, and the blood failing to receive the proper complement of oxygen taken in the respiratory tract, the arterial system would not have been duly filled, and the bluish pallor of the countenance would have passed into that deathly pallor which is seen in cases of bleeding from a large artery. The syphon system of the pulmonic circuit, which is always in action, would also have been endangered, and but for some secondary relief occurring under pressure, such as transudation of water into the pleural cavity, or transudation into the vesicular structure, there would have been death. These dangers were instantaneously removed by the letting of blood, and I am perfectly sure that if blood-letting were not one of the oldest of remedies, someone introducing it now for the first time in a few such cases as that I have named, would be considered one of the greatest pioneers of medical science. Unfortunately our forefathers when they bled were guided simply by symptoms, without regard to causes and conditions. They, therefore, fell into great error, because they did not differentiate between those symptoms in which the bleeding would be almost certainly useful, and corresponding symptoms, which might arise from precisely different conditions, in which bleeding would not be useful. In short, they transformed a particular into a general practice, and as they often blundered in the widespread and general method which they adopted, they brought a good practice into disrepute. Anyone who will read "The Practice of Medicine" of Dr. Armstrong will detect at once how great was the common error, and will be, for this reason, ready to put on the back shelf a book which, in many respects, is one of the shrewdest and most able in the English language.

